# Examining the Relationship Between Basic Psychological Needs and Athlete Identity During the COVID-19 Pandemic

**DOI:** 10.3389/fspor.2022.814301

**Published:** 2022-02-16

**Authors:** Patti C. Parker, Adam M. Beeby, Lia M. Daniels

**Affiliations:** Alberta Consortium for Motivation and Emotion, College of Social Sciences and Humanities, University of Alberta, Edmonton, AB, Canada

**Keywords:** athletes, COVID-19, basic psychological needs, athlete identity, motivation

## Abstract

**Objectives:**

During COVID-19 athletes have had games canceled, seasons postponed, and social supports lost. These changes negatively impact their motivation, and potentially identity, as athletes. We draw on self-determination theory to examine motivation in sport and its relationship with athlete identity during COVID-19.

**Design:**

A cross-sectional study design was employed consisting of online quantitative surveys.

**Method:**

We gathered background engagement and motivation data from 115 athletes involved in organized sport. They responded to questions on basic psychological needs satisfaction (competence, relatedness, autonomy) and athlete identity.

**Results:**

When reflecting on their basic psychological needs during the pandemic, most athletes considered them important. Athletes' competence and relatedness in sport were associated with social-related athlete identity, but not autonomy. Only relatedness in sport was associated with exclusivity-related social identity.

**Conclusions:**

Using a self-determination theoretical lens, our findings contribute to understanding athlete motivation and identities when sport is interrupted.

## Introduction

As a consequence of the COVID-19 pandemic, sporting events and competitions were put on hold for many individuals. This recent stoppage of sport is already negatively impacting athletes' mental health—including grief, stress, anxiety, depression, as well as their motivation in sport (Ruffault et al., 2020; Schinke et al., 2020). With the social isolation measures mandated by the pandemic, many athletes lost social supports, regular training regimens, and the ability to participate in their activities which are critical to their identities as athletes (Graupensperger et al., 2020). Athlete identity refers to the degree to which a person identifies with, and relates to, their role as an athlete (Brewer et al., 1993a) and is instrumental to commitment and investment in sport. How athletes' identities are threatened by the type of sport setbacks and interruptions brought about by COVID-19 is an important area for sport research. Our study investigates how the pandemic has affected athlete motivation and its relationship with athlete identity. We draw from self-determination theory (SDT) for a motivational lens to understand the athlete perspective during the pandemic.

SDT is defined as the internal development and inborn psychological needs that serve as foundational development toward a person's self-motivation, integration, and growth of the self (Ryan and Deci, 2002; Deci and Ryan, 2011). This type of motivation focuses on the self and is important for optimal functioning across various achievement domains, including school, sports, and career. Three basic psychological needs are identified in SDT: (a) *competence*, a person's sense of accomplishment or effectiveness within their environment; (b) *relatedness*, a person's connection with the people around them and in their community; and (c) *autonomy*, a person's feeling in control of their own behaviors (Ryan and Deci, 2002).

Competence, relatedness, and autonomy all matter because when they are met or experienced by a person they lead to internal growth resulting in greater self-motivation and mental health (Deci and Ryan, 2000). Whereas, when basic psychological needs are not met, they can result in less self-motivation and decreased wellbeing. It is not surprising that during the public health restrictions for COVID-19, across various countries athletes indicated having lower motivation (e.g., autonomous) (Martínez-González et al., 2021)—particularly expert athletes, those competing at a lower level, and those without training programs in place. Further, Leyton-Román et al. (2021) showed supporting athletes' need for competence (in Spain) and autonomous motivation helped bolster self-efficacy and commitment to practice. Additionally, athletes who reported more connectedness with teammates and greater social support during COVID-19 were better able to maintain their athlete identities (Graupensperger et al., 2020). Thus, we recognize the significance of self-motivation and athlete identities and focused on these relationships during the setbacks and interruptions associated with the COVID-19 pandemic.

Athlete identities can have many positive implications for athletes. For example, athlete identity is linked to enhanced athletic performance, commitment, and social connections (Brewer et al., 1993b; Horton and Mack, 2000). Notably, research on athlete identity reveals three dimensions that reflect social, exclusivity, and negative affectivity aspects of athlete identity (Brewer and Cornelius, 2001). Social-related athlete identity pertains to how much athletes view themselves as fitting the athlete role (e.g., “I consider myself an athlete”) and is associated with positive forms of personal competence such as social acceptance and behavioral conduct (Ryska, 2002), and athlete satisfaction (Burns et al., 2012). Exclusivity-related athlete identity reflects how much athletes have a narrow focus on sport and view themselves exclusively as an athlete (e.g., “I spend more time thinking about sport than anything else”). This dimension of athlete identity appears to benefit and harm aspects of motivation and wellbeing in that it is positively linked to scholastic and vocational types of competence (Ryska, 2002), but also burnout (Gustafsson et al., 2008) and is negatively related to athlete satisfaction (Burns et al., 2012). Finally, negative affectivity has to do with how athletes feel when they perform poorly or cannot fulfill their role as an athlete (e.g., “I feel bad about myself when I do poorly in sport”) and is related to athlete satisfaction (Burns et al., 2012) and anxiety (Masten et al., 2006).

For many, the athlete role can require a lot of commitment and investment, and lead athletes to feel a strong sense of who they are (Brewer et al., 1993b). However, when athletes' involvement in sport is threatened such as by injuries or early termination, they tend to reduce their identification, or dissociate from it, to avoid the costs of having to adjust (Lavallee et al., 1997; Brewer et al., 1999, 2010; Manuel et al., 2002). This dissociation can unfortunately lead to feeling a sense of loss and lower mental health (Sanders and Stevinson, 2017). These findings support the idea that the athlete identity is dynamic and can change, especially when threatened, and arguably, COVID-19 represents a global and ongoing threat to athlete identity. Schinke et al. (2020) further add that high-performance athletes place a lot of importance on their athlete identities and those at the highest levels who have made long-term commitments can have very focused and singular identities that may be incompatible with a socially isolating pandemic.

Since March 2020, competing athletes' engagement in their sport has been substantially, sometimes permanently, interrupted—this type of setback provides a unique situation in which to study athletes' self-determined motivation and how it relates to athlete identity. Thus, our study considers athletes' basic psychological needs during the COVID-19 pandemic and how needs satisfaction relates to two dimensions of athletic identity (social and exclusivity). Since we wanted to examine how their self-determined motivation impacts athletes' current views of their identity, for this study we were not interested in the third dimensions—negative affectivity—because it focuses on athletes' feelings of anticipated outcomes in sport which were largely paused.

Our study sought to understand athlete identity in the context of the COVID-19 pandemic. In doing so, as a first objective, we described athletes' reported frequencies of sport-related participation during the pandemic (e.g., how often they played their sport in the last 3 months). As a second objective, we examined the effect of autonomy, competence, and relatedness on social and exclusivity-related athlete identity while controlling for competitive sport status. We hypothesized that autonomy, competence, and relatedness would be positively associated with social-related athlete identity. This hypothesis is based on the idea that athletes who feel free to make choices, feel competent, and supported in their sport are likely to identify more with their social role as an athlete. Considering the conflicting evidence for exclusivity-related identity on motivation and wellbeing (Burns et al., 2012), we explored whether basic psychological needs would impact this type of identity but did not make specific hypotheses.

## Methods

We used a cross-sectional correlational design to collect self-report data on basic psychological needs and identity from adult athletes (18 years or older).

### Study Procedure

The study procedure received ethics approval from the researchers' institutional research ethics board (Pro00103711) and all participants provided informed consent. Data collection began at the beginning of December 2020 and finished at the end of January 2021. Participants were recruited using a snowballing method in which online advertisements (i.e., Twitter), listservs (i.e., Student Digest for a midwestern Canadian university), and emails were used to circulate the survey link and request that it be forwarded. A research assistant was responsible for regularly posting the recruitment materials (email, social media, university digests) and asking recipients to further share the opportunity for participation. The online survey link (SurveyMonkey) was included directly in the recruitment materials. Upon clicking the link, participants responded to a number of questions about sport participation (e.g., level of sport, sport type), demographic information (e.g., gender), and the target motivation and identity variables. There was no remuneration for participating. The survey took on average 11 min to complete.

### Participants

A total of 143 athletes clicked the survey link. Of these, the sample was reduced to 115 participants who met the study inclusion criteria of being athletes aged 18–25 who play at least one organized sport. We also excluded participants who had more than 30% missing data, indicating lack of full engagement with the survey. The sample consisted of 60% women, 37% men, and 3% were non-binary. The participants indicated playing the following sports: soccer (22), competitive dancing (12), volleyball (11), hockey (11), competitive cheerleading (9), weightlifting (7), basketball (5), team handball (5), multi-sport (4), distance running (4), rugby (4), ringette (3), swimming (3), alpine/cross-country skiing (2), football (2), golf (2), curling (1), track and field (2), ultimate frisbee (2) figure skating (1), gymnastics (1), paddling (1), and Jiu Jitsu (1). Of the sample, 41.7% comprised athletes playing organized sport at the competitive level, 31.2% at the recreational level, and 27.0% at both levels (see Table 1 for more sport-related descriptive information).

**Table 1:** Frequencies for background, sport engagement variables, and basic psychological needs.

	**Frequency**	**%**
1. Gender		
1 = Man	43	37.4
2 = Woman	69	60.0
3 = Non-binary	3	2.6
4 = Prefer not to share	0	–
2. Level organized sport is played		
1 = Recreational	36	31.3
2 = Competitive	48	41.7
3 = Both	31	27.0
3. Sport type		
1 = Team	14	12.2
2 = Individual	73	63.5
3 = Both	28	24.3
4. Competitive sport status (played competitively at some point)		
1 = No	13	11.3
2 = Yes	102	88.7
5. Amount sport was played pre-pandemic		
1 = More than once a week	84	73.0
2 = Weekly	29	25.2
3 = Every two weeks	2	1.7
6. Amount sport was played in last three months		
1 = More than once a week	33	28.7
2 = Weekly	17	14.8
3 = Every two weeks	3	2.6
4 = Once a month	9	7.8
5 = I didn't play this sport in the last three months	53	46.1
7. How long sport has been played		
1 = Age 2-5	29	25.2
2 = Age 6-12	36	31.3
3 = Age 13-18	28	24.3
4 = Started after high school	22	19.1
8. Able to return to sport as some regulations lifted during pandemic		
lockdown		
1 = No	52	45.2
2 = Yes	63	54.8
9. Autonomy: It is important for me to maintain my personal investment		
and commitment to training for my organized sport		
1 = Strongly disagree	2	1.8
2	0	–
3	7	6.3
4	6	5.4
5	12	10.8
6	14	12.6
7	6	5.4
8	12	10.8
9	11	9.9
10 = Strongly agree	41	36.9
10. Competence: It is important to me to have the ability to perform well		
in my organized sport once play is allowed to resume		
1 = Strongly disagree	2	1.8
2	0	–
3	3	2.7
4	1	0.9
5	6	5.4
6	9	8.1
7	12	10.8
8	12	10.8
9	12	10.8
10 = Strongly agree	54	48.6
11. Relatedness: It is important for me to maintain relationships with the people that are close to me within my organized sport (i.e., coaches, team)		
1 = Strongly disagree	0	–
2	1	0.9
3	3	2.7
4	4	3.6
5	8	7.2
6	6	5.4
7	17	15.3
8	20	18.0
9	15	13.5
10 = Strongly agree	37	33.3

*Frequencies shown for the demographic and sport engagement variables, and importance of basic psychological needs in sport during the COVID-19 pandemic*.

### Materials

The online survey consisted of a total of 46 items designed to measure participants' background information, basic psychological needs, and athlete identity.

#### Background Information

In order to get a sense of participants' previous and current sport engagement during the COVID-19 pandemic we asked seven general background questions such as their level of sport and if they had been able to return to their sport as COVID-19 regulations eased. This information was primarily collected to describe the sample. Descriptive information for each item including scales and frequencies are available in Table 1.

#### Basic Psychological Needs

We measured Basic Psychological Needs in two ways. First, we created three single-item measures asking participants to rate how important they felt satisfaction of competence, relatedness, and autonomy for sport is during COVID-19 (1 = *strongly disagree*, 10 = *strongly agree*). Second, we used Ng et al.'s (2011). Basic Needs Satisfaction in Sports Scale to measure participants' autonomy, competence, and relatedness as predictor variables. Participants were asked to answer these items while keeping in mind how they felt during the COVID-19 pandemic. All items were rated on a Likert scale (1 = *not true at all*, 7 = *very true*) and were slightly adapted to refer to sport as “organized sport”. Participants completed 4-items to measure their autonomy (e.g., “In my organized sport, I get opportunities to make choices”; Cronbach's α = 0.89), 5-items to measure their competence (e.g., “I get opportunities to feel that I am good at my organized sport”; Cronbach's α = 0.85), and 5-items to measure relatedness (e.g., “I have close relationships with people in my organized sport”; Cronbach's α = 0.81).

#### Athlete Identity

Athlete identity was examined as an outcome variable using Brewer and Cornelius' (2001) Athletic Identity Measurement Scale (AIMS; 1 = *strongly disagree*, 7 = *strongly agree*). The AIMS can be broken into three subscales reflecting social, exclusivity, and negative affectivity subscales (Brewer and Cornelius, 2001; Visek et al., 2008). The items were adapted to refer to sport as “organized sport”. Three items formed the social subscale measuring athlete's perceptions of how much they see themselves as an athlete and can maintain sport goals and relationships (e.g., “I consider myself an athlete”; Cronbach's α = 0.69). Two items formed the exclusivity subscale measuring athletes' perceptions on how focused and important their sport is in their lives (e.g., “I spend more time thinking about my organized sport than anything else”; Cronbach's α = 0.90). We chose not to analyze the third subscale measuring negative affectivity since, as previously mentioned, it reflected feelings on anticipated outcomes and not a current view of the athlete role.

### Analysis Plan

As a preliminary analytic plan, we employed descriptive analysis to assess frequencies of athletes' sport engagement in terms of sport level, sport type, competitive status, amount sport played pre-pandemic, amount sport played last 3 months, how long sport has been played, and whether athletes have been able to return to sport, as well as gender. In addition, we assessed frequencies of athletes' ratings of the importance of the three basic psychological needs during COVID-19 (see Table 1). Appropriate for a cross-sectional correlational design, we conducted zero-order correlations for the main study variables including athletes' competitive sport status, and basic psychological needs and athlete identity variables (see Table 2) before using OLS regressions to test if autonomy, competence, and relatedness explained a meaningful amount of variance in athlete identity in terms of social and exclusivity aspects (at *p* < 0.05). These regression tests covaried for competitive sport status. The results are reported using standardized regression coefficients (see Table 3).

**Table 2:** Zero-order correlation matrix and descriptive statistics for main variables.

	**1**	**2**	**3**	**4**	**5**	**6**
1. Competitive sport status	–					
2. Autonomy	−0.13	–				
3. Competence	0.31^*^	0.40^*^	–			
4. Relatedness	−0.09	0.50^*^	0.54^*^	–		
5. Athlete Identity (social)	0.29^*^	0.16	0.55^*^	0.52^*^	–	
6. Athlete Identity (exclusivity)	0.11	0.09	0.35^*^	0.35^*^	0.72^*^	–
*M*	1.89	21.84	29.97	30.07	16.05	7.30
*SD/%*	88.7%	5.22	4.48	4.72	3.92	3.74

*Zero-order correlations were calculated using pairwise deletion (n range = 105–115)*.

* *p < 0.01 (two-tailed tests). Competitive sport status was dummy-coded (1 = no, 2 = yes)*.

**Table 3:** Regression analyses: basic psychological needs and competitive sport status relationships with athlete identity.

	**Outcome variables**					
	**Athlete identity**					
	**Social-related**			**Exclusivity-related**		
**Predictor variable**	***β*** **(***b***)**	***t***	**95% CI of** ***β***	***β*** **(***b***)**	***t***	**95% CI of** ***β***
Competitive sport status	0.26^*^ (3.11)	3.15	1.15, 5.08	0.08 (0.87)	0.73	−1.51, 3.25
Autonomy	−0.15 (−0.11)	−1.70	−0.24, 0.02	−0.12 (−0.09)	−1.08	−0.24, 0.07
Competence	0.29^*^ (0.25)	2.88	0.08, 0.41	0.20 (0.16)	1.60	−0.04, 0.37
Relatedness	0.46^*^ (0.38)	4.80	0.22, 0.53	0.31^*^ (0.25)	2.61	0.06, 0.44
*AdjustedR* ^2^	0.44			0.14

*Standardized regression coefficients reported (unstandardized coefficients in brackets). Competitive sport status was dummy-coded (1 = no, 2 = yes)*.

* *p < 0.05*.

## Results

### Preliminary Analyses

All frequencies are presented in Table 1 and we highlight a few interesting patterns here. More than 80% of participants had been playing their sport since before they were in high school, with a quarter of respondents beginning in early childhood (age 2–5). A notable proportion of the sample played their organized sport competitively at some point (89%) and many played a team sport (64%). Before COVID-19, 73% of athletes indicated playing their sport more than once a week, a number that dropped to 29% over the course of the last 3 months of the COVID-19 pandemic. Moreover, 45% of participants said they had not played their sport at all since COVID-19 began in approximately March 2020. Between these two extremes, about 15% of participants had returned to play weekly, 3% were playing every 2 weeks, and 8% about once a month.

Regarding participants' perceptions of the importance of each basic psychological need, 89% agreed to strongly agreed competence is important in that they have the ability to perform well in their organized sport once play is allowed to resume. For relatedness, over 85% agreed it is important for them to maintain relationships with people close to them within their organized sport (i.e., coaches, team), whereas ~4% disagreed. For autonomy, 76% agreed to strongly agreed it is important to maintain their personal investment and commitment to training for their organized sport and <8% disagreed to some extent.

### Main Analyses

Zero-order correlations revealed positive associations between athletes' basic psychological needs of autonomy, competence, and relatedness in their organized sport during COVID-19 (*r*s = 0.40–0.54; see Table 2). Athletes' competitive sport status positively related to their reported competence and social-related athlete identity. Both their competence and relatedness positively correlated with social- and exclusivity-related athlete identities. Finally, social- and exclusivity-related athlete identities shared a strong correlation.

Our first OLS regression model comprised of autonomy, competence, and relatedness as predictor variables, and competitive sport status as a covariate, explained 44% of the variance in social-related athlete identity [*F*_(4, 99)_ = 21.50; *R*^2^ = 0.44, *p* < 0.001]. As expected, the analyses revealed both competence (*β* = 0.29, *p* < 0.001) and relatedness (*β* = 0.46, *p* < 0.001) were associated with social-related athlete identity. Autonomy was not significantly related (*β* = −0.15, *p* = 0.092). The model also revealed competitive sport status was positively associated with social-related athlete identity (*β* = 0.26, *p* < 0.001).

The second regression model was also significant which tested how much autonomy, competence, relatedness, and competitive sport status, explained exclusivity-related athlete identity [*F*_(4, 99)_ = 5.29; adjusted *R*^2^ = 0.14, *p* < 0.001]. In this model, we found only relatedness was positively associated with exclusivity-related social identity (*β* = 0.31, *p* < 0.001). Autonomy, competence, and competitive sport status were unrelated. The full model explained 14% of the variance in the outcome.

## Discussion

Our study focused on the relationships amongst the basic psychological needs of autonomy, competence, and relatedness and athlete identity during the COVID-19 pandemic. To address our study's first objective, we highlight a few important findings related to athletes' sport participation in the pandemic. To address our second objective, we discuss basic psychological need satisfaction and athlete identity in terms of social and exclusivity aspects. We conclude with implications, limitations, and directions for future research.

### Athletes in a COVID-19 Context

Before COVID-19, 73% of participants indicated they were playing their organized sport more than once a week prior to the pandemic, providing some confidence that this sample indeed reflected committed adult athletes. Remarkably only 29% reported having been able to return to that frequency of participation during the pandemic. Notably, just under half of the athletes had not been able to return to their sport in any form during the previous three months even as regulations began to lift. It is important to note that many of these athletes had been playing their sport since childhood, so lack of participation likely created a meaningful void in their lives. This finding reflects a loss of opportunity to engage in sport for athletes during the COVID-19 restrictions. Although this loss of opportunity can simply be assumed by the nature of public health restrictions, it shows that even as restrictions began to ease, not all athletes had been able to return to play—perhaps suggesting COVID-19 represents a setback or interruption that is less easily overcome than more traditional barriers such as a minor injury.

Athletes were much more likely to agree than disagree that satisfaction of basic psychological needs was important to their return to sport. Nearly 90% of participants reported competence as important, reinforcing the competitive and performance elements of sport. Likewise, relatedness with teams and coaches were viewed as important perhaps in response to the isolating and lonely reality many young people are experiencing in COVID-19 (Bu et al., 2020). Despite strong agreement for all needs, it is interesting that 25% of participants were neutral or disagreed about the importance of autonomy in terms of aspects such as personal investment and training. Again, it is possible that as COVID-19 progressed, athletes had to re-evaluate their personal commitments making it more important to examine how basic psychological needs relate to athlete identity.

### Athlete Identity

When considering social-related athlete identity—as in, how much athletes view themselves as athletes—our findings indicate both competence and relatedness positively relate to their athlete identity supporting our hypotheses. These results are both intuitive and align with research that suggests competence is linked to athlete identity reflections (Coatsworth and Conroy, 2009) and to behaviors—such as commitment to sport—that convey their athlete identities (Martínez-González et al., 2021). Feeling connected to others in sport is also strongly tied to social-related athlete identity (Graupensperger et al., 2020). Thus, it makes sense that the more competence athletes felt in their sport and the more connected, the stronger they viewed themselves as athletes, even during a pandemic. Furthermore, playing competitive sport was positively related to social-related athlete identity, which is consistent with Costa et al.'s (2020) study that found elite athletes, competing at higher levels, revealed higher athletic identities during lockdown.

Contrary to our hypothesis, our findings did not show autonomy was positively linked to social-related athlete identity. Although we cannot be sure, this may in part be explained by athletes' limited opportunities to make decision and choices in their organized sport as a result of the pandemic. This uncertainty about one's own volition in the sport experience may be one reason why athletes' autonomy is not related to their social-related athlete identity. Supporting this idea, recent evidence suggests university athletes experienced a significant decline in autonomous goal striving, as measured by identified and intrinsic motives, during their COVID-19 lockdown (Deci and Ryan, 2000). This study also highlighted that an athlete's resilience was a possible factor in helping to predict changes in autonomy over the lockdown period.

Athletes' levels of relatedness were positively associated with exclusivity-related athlete identities that reflected how focused they are on their organized sport. Autonomy, competence, and competitive sport status, however, did not predict this dimension of athlete identity. Of note, the bivariate correlation for competence and exclusivity-related athlete identity was significant but its influence on the identity outcome was suppressed when sharing variance with the other basic psychological needs. Evidently, during the pandemic, feeling a sense of belonging and support from others involved in sport (e.g., athletes and coaches) appears to promote a narrower focus on sport as conveyed by the exclusivity-related athlete identity measure. Athlete's perceptions of volition and how competent they felt in their organized sport did not seem to influence their focus on it. One speculation, is that since many athletes indicated engaging less frequently in their sport than before the pandemic, a sense of connection and support from important others (i.e., coaches and teammates) during the pandemic may have prompted them to acknowledge and re-focus on their sport role.

Our study also revealed findings concerning the role of competitive sport status for athletes' competence during the pandemic. Bivariate correlations between competitive sport status and competence and social-related athlete identity were significant. These findings imply that during COVID-19, playing at a more competitive level was associated with greater competence and social-related identity. Although seemingly intuitive, these associations reveal that the expected relationships still emerge during a global pandemic.

### Limitations and Implications

The results presented herein need to be considered in light of three limitations. First, athletes aged 18–25 years old were allowed to participate in the study but specific age and ethnicity information was not collected. Second, the sample is unbalanced in terms of gender with more women responding than men. Although these sample characteristics are less than desirable, there is little theoretical rationale for these demographic variables to influence basic psychological needs which have been shown to be universal principles (Ryan and Deci, 2008) and thus are unlikely to have substantially influenced our results. Nonetheless, more intentional recruitment strategies may produce more precise samples in future research. Third, this correlational study is only a snapshot of young adult athletes' participation in organized sports, basic psychological needs, and identity during COVID-19. More research is warranted to consider these findings using longitudinal study designs and addressing the context of the athlete population being examined.

Notwithstanding these limitations, the study findings are a timely and theory-based contribution to the literature on athletes' basic psychological needs and athlete identity during a global pandemic. In this circumstance, athletes may have fewer opportunities for autonomy not just in sport but in life in general, so it may be that competence and relatedness matter more concerning their social-related athlete identities. At the same time, athletes' relatedness, as in their social connectedness and support in sport during the pandemic, was related to a more myopic focus on their role as an athlete in sport.

Given that the majority of the athletes in our sample considered autonomy, competence, and relatedness important in their sport during COVID-19, this finding has implications for supporting athletes' basic psychological needs during critical setbacks and sport interruptions. A qualitative study by Bejar et al. (2019) found athletes enduring an injury were overall motivated in their sport injury rehabilitation when they perceived their athletic therapists to be fulfilling these needs. Another study found that depending on athletes' needs being satisfied or frustrated had an impact on their wellbeing during the COVID-19 outbreak (Šakan et al., 2020). Additionally, Leguizamo et al. (2021) studied the confinement of athletes during the pandemic and found that employing coping strategies, such as “emotional calming” and “cognitive restructuring” were negatively associated with negative emotional states such as anxiety, depression and stress. These studies point to the importance of equipping athletes with trained personnel, resources, and cognitive coping strategies, in order to support their basic psychological needs during sport setbacks.

Specifically, our findings suggest sport professionals (sport psychologists, coaches, etc.) may be able to help foster social-related athlete identities during COVID-19 and other sport interruptions by focusing on strategies to enhance competence (e.g., giving athletes goals and skills to work on) and relatedness (e.g., creating spaces to strengthen relationships with teammates and staff via digital platforms). To combat possible negative outcomes that can result from strong exclusivity-related athlete identities (Gustafsson et al., 2008), athletes can be encouraged to seek out valued support in other places, such as family, career or school, particularly to help them adjust in times when opportunities for sport engagement is limited. Furthermore, our findings have implications for education programs. Physical education programs in schools and communities can benefit from understanding how athletes' basic psychological needs can impact their athlete identities so that individuals working with athletes can learn ways to satisfy these needs during unprecedented times.

## Conclusion

Our study showed that the majority of young adult athletes valued basic psychological needs of autonomy, competence, and relatedness during the COVID-19 pandemic. With athletes having fewer opportunities for autonomy in sport during the pandemic, competence and relatedness appeared to matter more in terms of their social-related athlete identity—while relatedness during the pandemic was related to a greater focus on exclusivity-related athlete identity. Our findings point to helping athletes foster adaptive sport identities during COVID-19 by encouraging strategies to build competence and relatedness; while reminding athletes of other places to seek connectedness when sport engagement opportunities are limited.

## Data Availability Statement

The raw data supporting the conclusions of this article will be made available by the authors, without undue reservation.

## Ethics Statement

The studies involving human participants were reviewed and approved by University of Alberta Research Ethics Board 2. The patients/participants provided their written informed consent to participate in this study.

## Author Contributions

PP, LD, and AB contributed sufficiently to the manuscript to justify authorship. AB and LD conceptualized the project, defined the methodology, and were involved in the data collection. PP and AB conducted the analysis and drafted the original manuscript. All authors were involved in the interpretation and write-up of the results, contributed to the final version and approval of the manuscript, and agreed to be accountable for the content of the work.

## Funding

This study was supported by a research grant to LD from the Social Sciences and Humanities Research Council of Canada (RES0024734).

## Conflict of Interest

The authors declare that the research was conducted in the absence of any commercial or financial relationships that could be construed as a potential conflict of interest.

## Publisher's Note

All claims expressed in this article are solely those of the authors and do not necessarily represent those of their affiliated organizations, or those of the publisher, the editors and the reviewers. Any product that may be evaluated in this article, or claim that may be made by its manufacturer, is not guaranteed or endorsed by the publisher.
